# Effects of Graphene Oxide and Chemically-Reduced Graphene Oxide on the Dynamic Mechanical Properties of Epoxy Amine Composites

**DOI:** 10.3390/polym9090449

**Published:** 2017-09-14

**Authors:** Cristina Monteserín, Miren Blanco, Estibaliz Aranzabe, Ana Aranzabe, Jose Manuel Laza, Aitor Larrañaga-Varga, Jose Luis Vilas

**Affiliations:** 1 Unidad de Química de Superficies y Nanotecnología, Fundación Tekniker, Iñaki Goenaga 5, 20600 Eibar, Spain; miren.blanco@tekniker.es (M.B.); estibaliz.aranzabe@tekniker.es (E.A.); ana.aranzabe@tekniker.es (A.A.); 2 Departamento de Química Física, Facultad de Ciencia y Tecnología, Universidad del País Vasco/EHU, Apdo. 644, E-48080 Bilbao, Spain; josemanuel.laza@ehu.eus (J.M.L.); joseluis.vilas@ehu.eus (J.L.V.); 3 SGIker, General Research Services, University of the Basque Country (UPV/EHU), B. Sarriena S/N, 48940 Leioa, Spain; aitor.larranaga@ehu.eus

**Keywords:** thermosetting resin, graphene, nanocomposites, thermomechanical

## Abstract

Composites based on epoxy/graphene oxide (GO) and epoxy/reduced graphene oxide (rGO) were investigated for thermal-mechanical performance focusing on the effects of the chemical groups present on nanoadditive-enhanced surfaces. GO and rGO obtained in the present study have been characterized by Fourier transform infrared spectroscopy (FTIR), X-ray photoelectron spectroscopy (XPS), and X-ray powder diffraction (XRD) demonstrating that materials with different oxidation degrees have been obtained. Thereafter, GO/epoxy and rGO/epoxy nanocomposites were successfully prepared and thoroughly characterized by dynamic mechanical thermal analysis (DMTA) and transmission electron microscopy (TEM). A significant increase in the glass transition temperature was found in comparison with the neat epoxy. The presence of functional groups on the graphene surface leads to chemical interactions between these functional groups on GO and rGO surfaces with the epoxy, contributing to the possible formation of covalent bonds between GO and rGO with the matrix. The presence of oxidation groups on GO also contributes to an improved exfoliation, intercalation, and distribution of the GO sheets in the composites with respect to the rGO based composites.

## 1. Introduction

Epoxy systems are one of the most important organic matrices in the composite industry, frequently used in demanding applications, such as matrices in reinforced composites, adhesives in the aerospace industry, surface coatings, etc., due to their excellent mechanical properties, high chemical and thermal stability, excellent adhesive properties, good corrosion resistance, and low shrinkage during the curing process. However, their brittleness, poor resistance to crack propagation and poor wear resistance limit their applications. A large number of studies have been published in order to improve the toughness of epoxy composites. Different types of fillers, such as rubbers, thermoplastic materials, or inorganic nanoparticles with different chemical natures, have been added to the epoxy systems as a third component to ensure a remarkable enhancement in the mechanical and thermal properties [1,2,3,4]. The incorporation of rubber or thermoplastics into the epoxy systems form a multi-phase structure with a discrete second phase can improve fracture toughness, but in most cases, with reductions in the modulus and the glass transition temperatures (*T*_g_) or involving a high cost [5]. Inorganic particles, such as silica and alumina, with diameters between 4 and 100 μm, have been used to increase the toughness of epoxies without sacrificing their basic properties, but the presence of numerous and relatively large inorganic particles increases the viscosity leading to poor dispersion and processing difficulties [6,7].

To overcome these problems, the addition of a nanophase structure in the epoxy matrix has been studied. Various parameters, i.e., the type of nanoadditive, size, shape, and the nanoadditive/epoxy matrix interfacial cohesion can influence the mechanical properties of a polymer [8]. Between the different nanoparticles, the use of more flexible and tough carbon nanomaterials, such as carbon nanotubes (CNTs), graphene, and carbon nanofibers has been shown to further increase the toughness of the epoxy polymer [9,10,11,12,13,14]. 

The enhancement of mechanical properties on polymeric materials by the incorporation of graphene based materials shows a higher extent to improve their properties due to the high aspect ratio and excellent properties of graphene. Graphene presents a thermal conductivity around 5000 W/m·K, an electrical conductivity of 6000 S·cm^−1^, and as a monolayer, presents a high specific surface area (2630 m^2^/g). Furthermore, graphene presents excellent mechanical properties, a Young’s modulus of almost 1100 GPa, anda tensile strength of approximately 125 GPa, 200 times higher than steel [15,16,17,18]. 

The advantages of polymeric nanocomposites derived from the presence of the exfoliated morphology of graphene also include a higher glass transition temperature, stiffness, and strength due to polymer chain confinement effects resulting from the tremendous surface area developed. However, the improvement in the physicochemical properties of epoxy nanocomposites depends on the distribution of graphene layers in the polymeric matrix, as well as the interfacial bonding between the graphene layers and the polymeric matrix. Pristine graphene is not compatible with organic polymers and does not form homogeneous composites. Therefore, the agglomeration of graphene has been one of the main barriers limiting its potential use as a mechanical reinforcing agent. In contrast, graphene oxide (GO) sheets are highly-oxygenated graphene containing different functional groups, such as epoxy, alcohol, ketone, carboxylic, and ether, and/or the presence of debris that can alter the van der Waals interactions significantly, making them more compatible with organic polymers [19,20,21]. For this reason recent research has stimulated considerable interest in the field of graphene oxide/polymer nanocomposites [3,16,22,23,24,25,26,27,28,29,30].

In order to increase the dispersion of graphene in different chemical environments, a large number of routes of chemical modification of its surface have been proposed [24,31,32]. Many strategies were based on initial oxidation processes [16,33]. The electrical conductivity of the resulting nanocomposites can be increased by the chemical reduction of GO, presumably by restoring the graphitic network of sp^2^ bonds.

Understanding the curing behavior of epoxy systems is essential because the thermal and mechanical properties of the epoxy composites depend on the formation of the crosslinked molecular network in the system and the structure of the interphase region between the continuous phase (resin) and on the discontinuous phase (reinforcement). In a previous research, the curing kinetics of epoxy nanocomposites prepared by incorporating graphene oxide and reduced graphene oxide (rGO) have been studied using isothermal and non-isothermal differential scanning calorimetry [34]. The GO load in epoxy amine systems resulted in a decrease of peak exotherm temperature and a reduction in the curing times, indicating a significant enhancement of curing reaction. For the same curing temperature, the addition of GO resulted in a great acceleration of the curing process; however, the reaction rate was barely modified with the inclusion of rGO.

The aim of this study is to analyze the effect of reduced graphene oxide and graphene oxide addition on the thermal-mechanical properties of epoxy-amine systems. The study focuses on chemical interaction between functional groups on GO and rGO surfaces with the epoxy-amine system and the effects of these interactions on thermal-mechanical properties and on the exfoliation, intercalation, and distribution of the nanoadditives in the epoxy composites. For this purpose, the viscoelastic properties of the nanocomposites have been investigated by dynamic mechanical analysis (DMTA) and the distribution of GO and rGO on the composites by X-ray powder diffraction (XRD) and transmission electron microscopy (TEM).

## 2. Materials and Methods

### 2.1. Materials

The epoxy resin employed in this study was a difunctional diglycidyl ether of bisphenol A (DGEBA) with an equivalent weight of 175–180 g/equiv. and a hydroxyl/epoxy ratio of 0.03, supplied by Sigma-Aldrich (St. Louis, MI, USA). The hardener was a solid tetrafunctional aromatic amine, 4,4′-diaminodiphenylmethane (DDM), from Sigma-Aldrich, with a molecular weight of 198 g/mol and an amine equivalent weight of 49.5 g/mol. 

Commercial powder graphite containing 99 wt % minimum of carbon, 0.8 wt % maximum of ash, and 0.05 wt % maximum of sulfur from Acros Organic was selected for the preparation of GO and rGO. For the oxidation of graphite, sodium nitrate was purchased from Acros Organic, whereas sulfuric acid (95–97%), potassium permanganate (extra pure) and hydrogen peroxide (30 wt %) were purchased from Scharlau. For the GO reduction, monohydrated hydrazine (64–65%) from Sigma Aldrich was used. Pure water by ELIX Advantages (type II) was employed in the current work. All reagents have been used without further purification.

### 2.2. Sample Preparation

#### 2.2.1. Synthesis of GO and rGO

Among the different chemical routes that already exist for the production of powder graphene, it has been used a modified Hummers and Offeman method [35] followed by a chemical reduction with hydrazine. 

Graphene oxide (GO) was prepared by completely oxidizing natural graphite flakes. Five grams of graphite powder were added into 115 mL of concentrated sulfuric acid (H_2_SO_4_) in a 1 L reaction vessel. The mixture was maintained at 10 °C. Thereafter, a mixture of 15 g of potassium permanganate (KMnO_4_) and 2.5 g of sodium nitrate (NaNO_3_) was added gradually with stirring maintaining the temperature of the mixture below 20 °C. The temperature was raised to 35 °C and the mixture was maintained at 35 °C for 30 min. Next, 140 mL of water was added dropwise (10 mL/min) giving an increase in temperature to 98 °C, and the mixture was maintained at that temperature for 30 min. Then, 250 mL of distilled water were added to the solution followed by 25 mL of 30 wt % hydrogen peroxide (H_2_O_2_) solution to finish the reaction. After cooling, the sample was sonicated for 15 min at room temperature and the solid product was separated by centrifugation, washing the sample repeatedly with a 5 wt % HCl solution to remove traces of sulphates and with distilled water to remove permanganate residues. Finally, the resulting GO sample was dried overnight at 80–85 °C under vacuum.

The GO reduction was achieved by adding hydrazine, 1 µL of hydrazine per 1 mL of water, and maintaining the mixture under stirring for 8 h at 80 °C [35,36]. According to the theory, a black precipitate appears, as the oxygen group content in rGO is lower than in GO, and rGO become hydrophobic. The process was completed when the rGO was cleaned with distillated water to remove residual hydrazine and it was dried overnight under vacuum at 80–85 °C [37].

#### 2.2.2. Preparation of Modified Epoxy Amine Composites

Composites with 0.5, 2 and 5 wt % of GO and rGO were prepared by mixing mechanically the nanofillers with the epoxy resin and then sonicating the system at room temperature for 30 s. The dispersions were mixed with the hardener in an epoxy-amine stoichiometric ratio at 80 °C stirring vigorously for 5 min. The mixtures were transferred to glass molds and cured at 90 °C during 4 h.

### 2.3. Characterization Methods

#### 2.3.1. Fourier Transform Infrared Spectroscopy (FTIR)

Fourier transform infrared spectroscopy has been employed for the characterization of the nanoadditives used in the current study. FTIR spectra were obtained using a FT/IR 4700 (JASCO, Easton, MD, USA) system to study the functional groups that appear in the oxidation process and the elimination of these groups in the reduction. The GO and rGO solid samples were pulverized and mixed with KBr. Then, the mixture was compressed into pellets. Twenty scans were taken for each sample with a resolution of 8 cm^−1^.

#### 2.3.2. X-ray Photoelectron Spectroscopy (XPS)

XPS measurements were carried out for the characterization of GO and rGO. XPS was performed with a SPECS system (Berlin, Germany) equipped with a Phoibos 150 1D-DLD spectrometer and Al Kα (1486.7 eV) monochromatic radiation source.

An initial analysis of the present elements was carried out (wide scan: step energy 1 eV, dwell time 0.1 s, pass energy 80 eV) and detailed analyzes of the elements were performed with an electron output angle of 90° (detail scan: step energy 0.1 eV, dwell time 0.1 s, pass energies of 30 and 100 eV). The spectra were adjusted using CasaXPS 2.3.16 software (Wilmslow, Cheshire, UK), which models Gauss-Lorentzian contributions, after a subtraction of the background (Shirley).

#### 2.3.3. X-ray Powder Diffraction (XRD)

X-ray diffraction patterns were performed on the samples of graphite, GO, and rGO as well as on the composites prepared containing 0.5, 2, and 5 wt % of GO and rGO. The X-ray powder diffraction patterns for identification and signal deconvolution of the maxima were collected by using a X’PERT PRO automatic diffractometer (PANalytical, Almelo, Netherlands). The operating conditions were 40 kV and 40 mA, in theta-theta configuration, secondary monochromator with Cu Kα radiation (λ = 1.5418 Å) and a PIXcel solid state detector.

Texture evaluation of the composite samples was measured using a Bruker D8 Discover diffractometer (Karlsruhe, Germany) equipped with a Cr Twist tube, Ni filter (λ = 2.298 Å), PolyCapTM (1μ single crystal cylinders) system for parallel been generation (divergence of 0.25°), and a 1-D LynxEye detector (active length in 2θ 2.7°). The sample was mounted on an Eulerian cradle with an automatically controlled X-Y-Z stage. Data were collected for the (002) reflection at 39.32° in 2θ (using a fixed mode and time per orientation of 80 s). The data collection in standard mode with 5° of d was measured for full circle 0–360 in increments of 5° in Phi (φ) and 0–75 in increments of 5° in the Psi (ψ) range, giving 693 total orientations.

Texture analysis using X-ray diffraction has typically been performed via the use of pole figure measurement. Such measurements were performed by measuring exact 2θ maxima and rocking the sample through Psi (tilt) angles and Phi (spindle) rotations via a texture cradle attachment. The measured intensities are then plotted as an intensity map where the hemisphere-like distribution of scattered intensity is projected on a 2D “pole figure” showing the variation of intensity with the sample orientation. 

#### 2.3.4. Transmission Electron Microscopy (TEM)

TEM images were performed on the samples of composites prepared containing 0.5 wt % of GO and rGO. The films were sectioned using an ultramicrotome device at room temperature (Leica EMUM 6) equipped with a diamond knife. The ultrathin sections (~100 nm) were placed on a 300 mesh copper grid. The grids were examined in a transmission electron microscope, TECNAI G2 20 TWIN (FEI, Hillsboro, OR, USA), operating at an accelerating voltage of 200 KV in a bright-field image mode. 

#### 2.3.5. Dynamic Mechanical Thermal Analysis (DMTA)

DMTA measurements were performed on the samples of composites prepared containing 0.5, 2, and 5 wt % of GO and rGO. A Polymer Laboratories Mark II DMTA instrument (Agilent Technologies, Santa Clara, CA, USA) was used to analyze the composites. Rectangular samples (10 mm × 35 mm × 1.5 mm) were directly cut from the composite sheets and measured using the dual cantilever test from −50 °C to 250 °C, at a heating rate of 2 °C/min, 64 µm strain, and at several deformation frequencies (1, 3, and 10 Hz). Samples of the neat epoxy system were also measured under the same conditions.

## 3. Results and Discussion

### 3.1. Characterization of Carbon Based Nanoadditives

Graphene oxide sheets were prepared from natural graphite powder with a modified Hummer Offeman oxidation method. Thereafter, reduced graphene oxide sheets were prepared by its chemical reduction with hydrazine. The oxidation of graphite and the reduction of graphene oxide were checked by FTIR, XRD, and XPS. 

**Figure 1:**
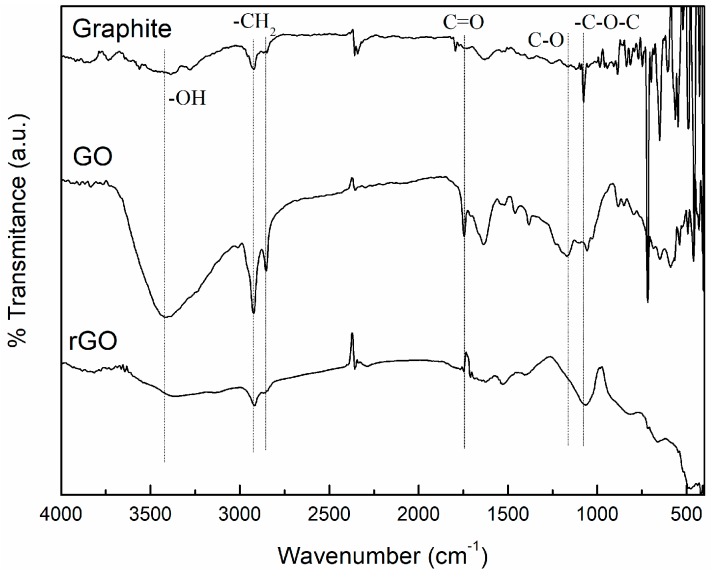
FTIR spectra of graphite, GO, and rGO.

Figure 1 shows the FTIR spectra of GO and rGO. The spectra corresponding to the commercial graphite initially employed for its preparation has been also included for comparison purpose. While no significant peaks were observed in raw graphite, graphene oxide was found to exhibit several characteristic absorption bands of oxygen-containing groups, demonstrating that the oxidation process was successful. A broad peak at 3400 cm^−1^ appeared for the O–H stretching vibrations from hydroxyl groups and water molecules. The IR peaks corresponding to 2927 cm^−1^ and 2849 cm^−1^ are due the asymmetric and symmetric CH_2_ stretching of GO. These bands are also present in natural graphite due to the presence of impurities or chemical defects in the sp^2^ graphitic plane. Some degree of deformation in the hexagonal structure of graphite sheet is also observed below in XRD analysis. The intensity of these bands is higher for GO due to the presence of more defects on the structures and, therefore, to the presence of a higher amount of C–H vibrations. Then, the vibrations for C=O stretching and C–O stretching of –COOH groups generated at edges of the GO sheet’s surface appeared at 1743 cm^−1^ and 1210 cm^−1^, respectively. Some bands also appear around 1050 cm^−1^ which can be ascribed to the generation of C–O–C stretching vibrations of epoxy groups. It can be observed that the intensities of absorption bands of oxygen-containing functional groups were dramatically reduced for the rGO. The carbonyl C=O bands were found to have disappeared, but it can be seen that the spectrum retains a broad absorption band centered at 3400 cm^−1^ and presents a new band around 1050 cm^−1^ which was attributed to the residual O–H groups in rGO. The presence of a higher content of oxidation groups on GO has been also determined by thermogravimetric analysis (TGA) in a previous study [33].

**Figure 2:**
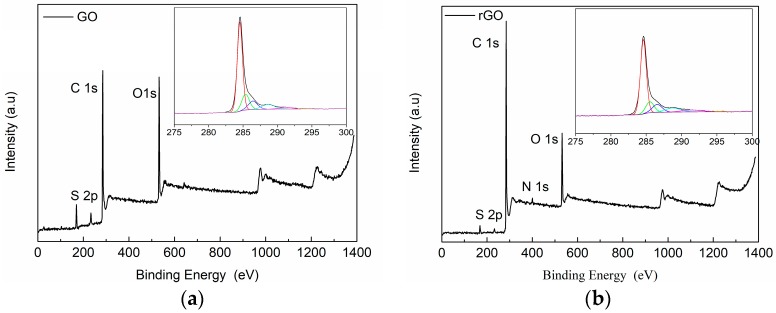
High-resolution XPS spectra of: (**a**) GO and (**b**) rGO.

Moreover, XPS and XRD were also employed to analyze the structure of GO and rGO. XPS is a surface-sensitive analytical technique that is useful to determine the chemical environment of atoms. The XPS spectra were analyzed to further identify the chemical composition of the surfaces and possible changes in the surface characteristics of the GO and rGO samples. Figure 2 shows the XPS spectra of GO and rGO. A magnification of the C1s XPS spectra of GO and rGO samples are also included in the framework of Figure 2a,b. Table 1 provides an analysis of the spectrum peaks, their binding energies and atomic percentage of each group. The contents of C, O, and S elements in GO were 69.5%, 25.2%, and 5.3%, respectively, while the contents of C, O, N, and S elements in rGO were 81.4%, 15.6%, 1.2% and 1.7%, respectively. C1s XPS spectra of GO indicates considerably degree of oxidation that means the presence of different oxygen functional groups in the GO structure (e.g., carbonyl, epoxy, hydroxyl groups). The spectrum of the GO sample presented different deconvolution peaks, corresponding to the C–C/C–H in aromatic rings (284.6 eV), epoxy C (C–O, 286.6 eV) and carboxyl C (–COOH, 288.9 eV), respectively. The C1s XPS spectrum of rGO also exhibits these oxygen functional groups, indicating the presence of similar C–O links in this sample, however, a clear reduction is observed in the O content in rGO samples, as can be seen by the ratio between the signal of C1s and O1s. These results suggest significant removal of oxygen functional groups in rGO. The values of relative at % in Table 1 also show the dropping of O content from 25.2% for GO to 15.6% for rGO, ascribed to the successful reduction of GO.

**Figure 3:**
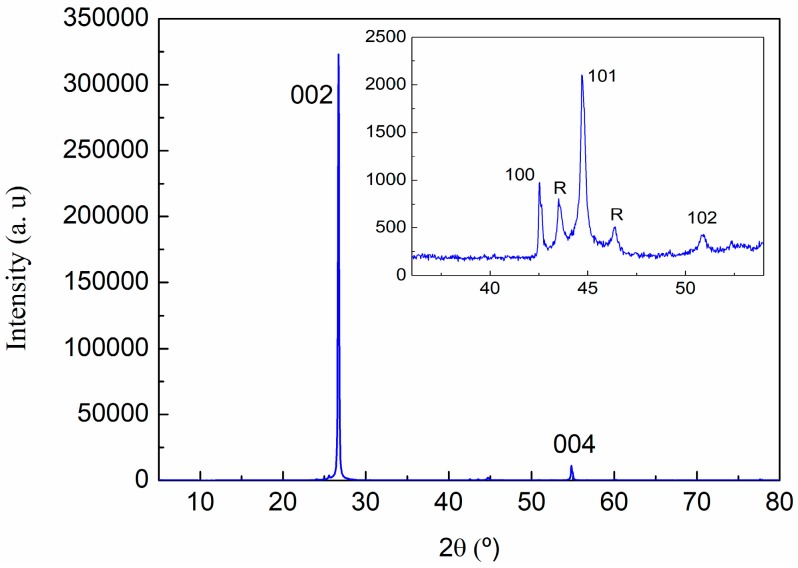
X-ray diffraction pattern of graphite with the assigned (*hkl*) Miller indices.

**Figure 4:**
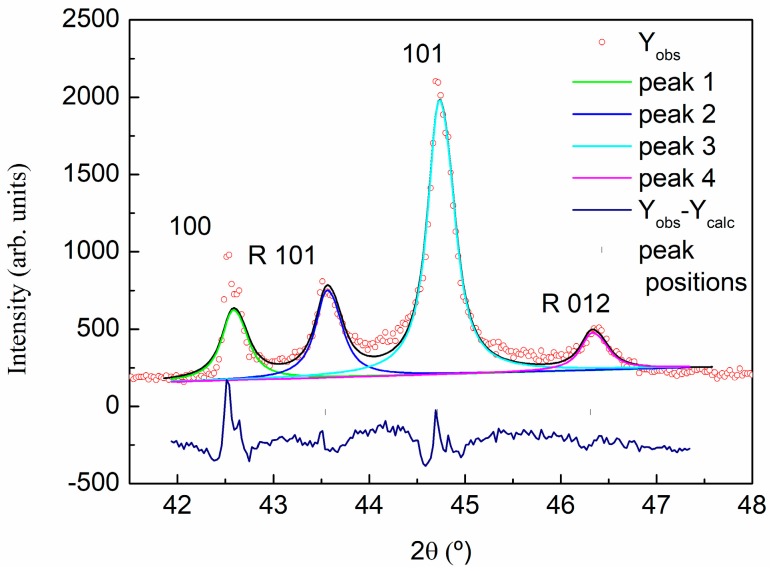
X-ray diffraction pattern of graphite. Profile refinement and the deconvolution of 41.5–47.5° 2θ range of the pattern.

For the XRD analysis of graphite, GO, and rGO, a preliminary identification of the diffraction peaks was evaluated using the Powder Diffraction File (PDF) database. The graphite sample was assigned to 00-041-1487 reference hexagonal graphite, *P*63/*mmc* (194) space group. As was expected, the diffraction pattern of the graphite is dominated by the strong (002) reflection at 2θ = 26.70° (see Figure 3 and Table 2). A deconvolution of the peak in the 2θ range of 40–49° shows the contribution of the graphite (100) and (101) reflections in the case of the hexagonal graphite, and (101) and (012) reflections of the rhombohedral symmetry phase (Figure 4), indicating some degree of deformation in the hexagonal structure of graphite sheets. Measured interplanar spacings converted to lattice constants for graphite, shown in Table 2, indicate an interlaminar distance around 3.34 Å.

**Figure 5:**
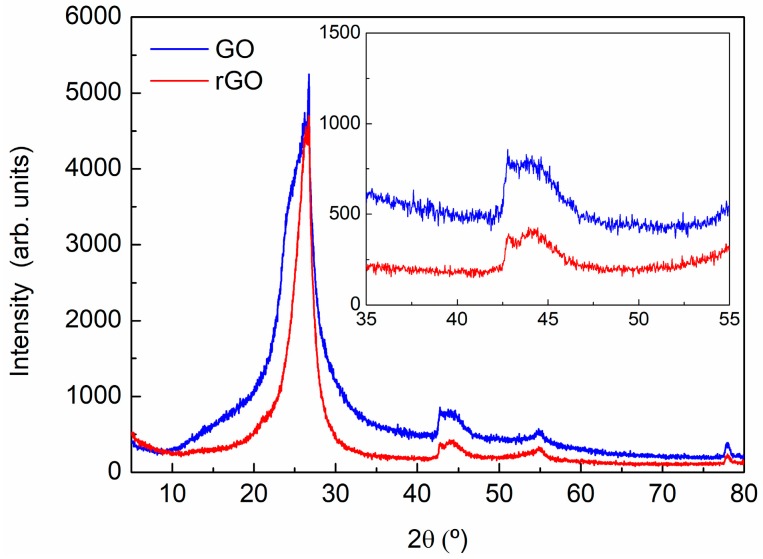
X-ray diffraction pattern of GO and rGO.

In the GO and rGO X-ray diffraction pattern, Figure 5, an intense broadening, low-angle displacement, and reduction of the punctual intensity of the maxima corresponding of the initial graphite is shown, indicating larger interplanar spacing and lower crystallinity.

**Figure 6:**
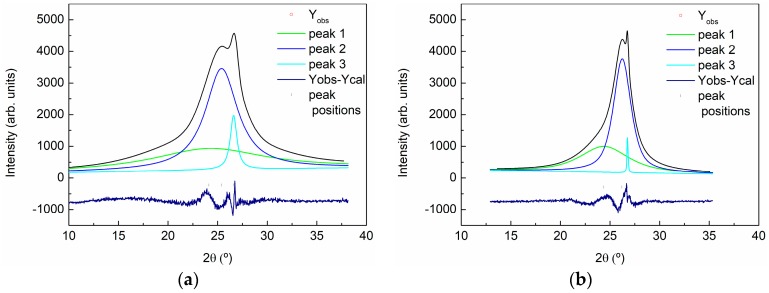
X-ray diffraction pattern of GO and rGO. Deconvolution of 10–40° 2θ range of the pattern of GO (**a**) and rGO (**b**).

The deconvolution of the signal for GO and rGO can provide the exact position and half-width parameters of the signals. This procedure was carried out using the peak-fit option of the WinPLOTR program (T. Roisnel (CEA/CNRS), J. Rodriguez-Carvajal (ILL), France) without a structural model. The simulated profile was shown in Figure 6. The measured interplanar spacings for both samples were converted to lattice constants and are shown in Table 3.

The deconvolution of the peak showed the presence of three mean crystallinities in the materials. The peak appearing at higher position indicates the presence of a small fraction (<5 wt %) of residual graphite in both samples. The appearance of the other two peaks, which did not appear for graphite, indicated an increase in the interplanar distance and a loss in the spacing periodicity (taking into account the planarity of the layers). This sheet’s dispersion is a consequence of the existence of oxygen-rich groups on both sides of the sheets and water molecules trapped between the sheets. These peaks appeared at lower 2θ values than for GO with respect to rGO, and the full width at half maximum of the peak (FWHM) increased, indicating a higher presence of disorder on GO. On the other hand, the low intensity and the high broadening in the 2θ 40–48° range, makes impossible the deconvolution of the signal, being a consequence of the oxidation treatment that affects the hexagonal structure of the layers. 

Summarizing, FTIR, XPS, and XRD results showed the presence of different oxygen functional groups in the GO structure (e.g., carbonyl, epoxy, hydroxyl groups) confirming the successful oxidation of GO. Moreover, a significant removal of oxygen functional groups in rGO was obtained, indicating a partial reduction of the functional groups. 

### 3.2. Characterization of GO- and rGO-Based Epoxy Composites

After the characterization of the nanoadditives, composites with 0.5, 2, and 5 wt % of GO and rGO were fabricated by mixing GO and rGO with epoxy resin and curing the different systems at 90 °C for 4 h. The composites have been characterized by XRD, DMTA, and TEM. 

**Figure 7:**
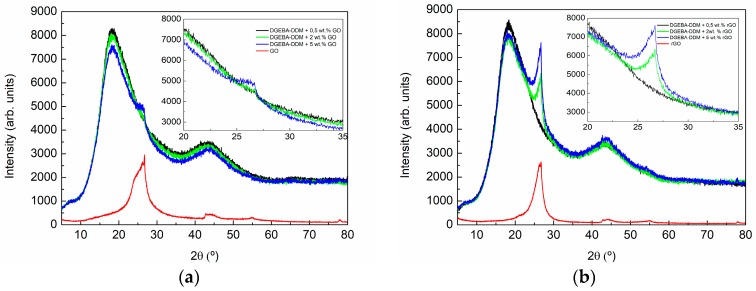
X-ray diffraction pattern of the composites with 0.5, 2, and 5 wt % of: (**a**) GO and (**b**) rGO. The right part shows a magnification of 20–35° 2θ range of the patterns.

Figure 7 shows the XRD pattern of the composites with 0.5, 2, and 5 wt % GO (Figure 7a) and rGO (Figure 7b). A wide diffraction appeared in all patterns from 5° to 80° with two maxima around 20° and 45° caused by the scattering of the cured epoxy network and revealing its amorphous nature. A third peak maximum appears as a shoulder in the peak centered around 20°. A magnification of this region can be shown in the right part of Figure 7a,b. This peak increases in intensity with the increase in GO and rGO content. In both type of composites small amounts of GO and rGO are not distinguishable, however, quantities higher than 2 wt % of GO can be clearly observed. Comparing the patterns obtained for composites with the same content of GO and rGO, the intensity of the peak is lower for GO modified composites than for rGO modified composites, even if the intensity of this signal is similar for both nanomaterials (see Figure 5). The reduction in intensity indicates that the volume of the sample that is able to diffract is lower for GO-modified composites and this may be an indication that a better exfoliation of GO sheets in composites is being obtained. For both types of composites, a broadening of the shoulder toward lower angles is observed. The effect is more evident for the GO-modified composites. This could indicate that a better intercalation of GO sheets in the epoxy matrix occurred as compared to rGO.

**Figure 8:**
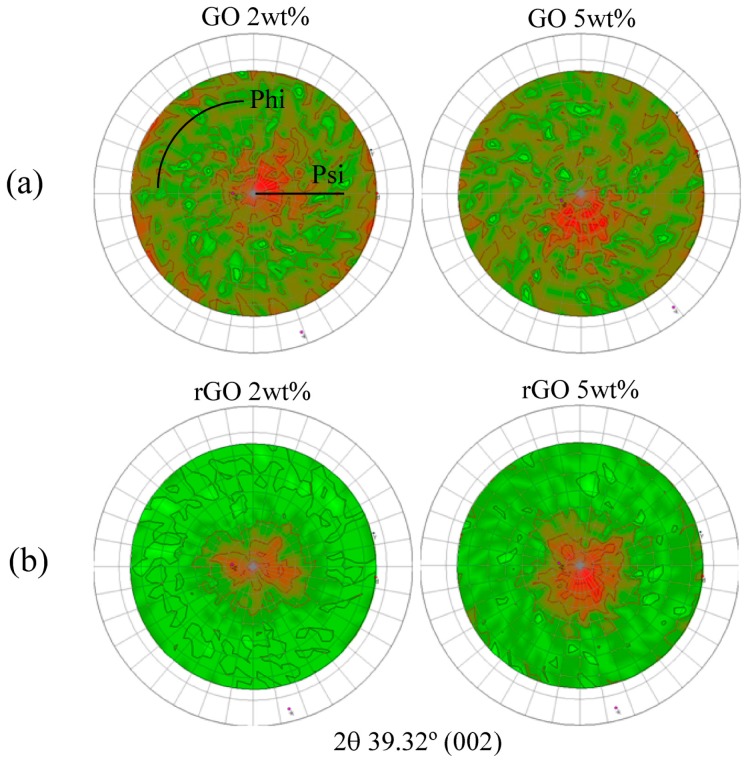
Pole figures of the composites with 2 and 5 wt % of: (**a**) GO and (**b**) rGO in the perpendicular plane.

In Figure 8, the distributions of crystallographic orientations of the composites with 2 and 5 wt % GO and rGO are shown. The pole figures for all composites showed a preferential orientation of GO and rGO. However, the samples modified with GO showed a more heterogeneous structure with small amounts of layers diffracting in every direction, as can be seen from the higher distribution of the red area in the images. 

**Figure 9:**
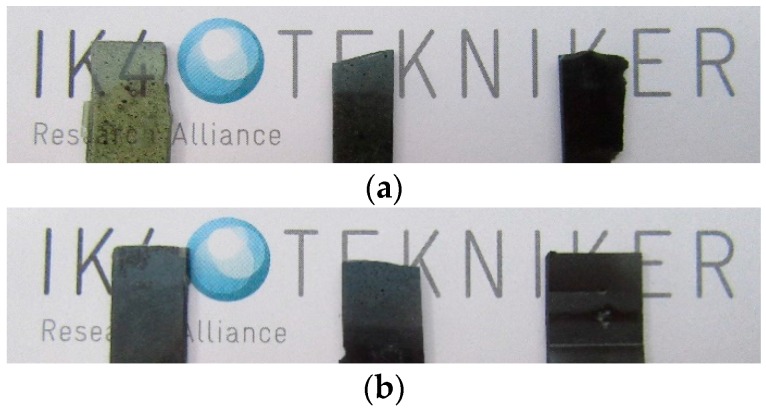
Final appearance of composites with 0.5, 2, and 5 wt % of: (**a**) GO and (**b**) rGO.

The better exfoliation, intercalation and distribution of the GO sheets in the composites with respect to the rGO can be responsible of the higher transparency observed for GO-modified composites (see Figure 9). 

**Figure 10:**
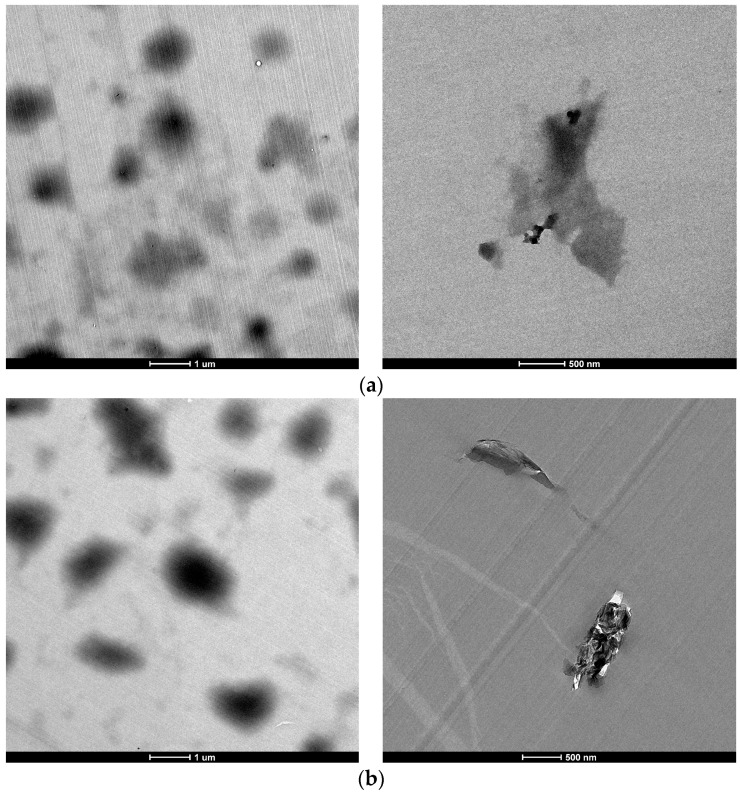
TEM images of epoxy/graphene composites with 0.5 wt % of: (**a**) GO and (**b**) rGO.

The morphology of the epoxy composites containing 0.5 wt % of GO and rGO was examined by TEM (Figure 10). Even if some large agglomerates are distinguished optically (Figure 9), at the microstructural level, it can be seen that GO and rGO nanosheets are individually and randomly dispersed in the epoxy matrix without significant aggregation. The rGO nanosheets showed increased thickness (higher opacity in the image) and lower exfoliation in comparison with the GO nanosheets. GO nanosheets seem to be more individual. This fact is in agreement with the presence of oxidation groups on GO sheets which contributes to an improved exfoliation, intercalation and distribution of these sheets in the composites. This is also reflected in the transparency observed in the epoxy composites with 0.5 wt % of GO.

**Figure 11:**
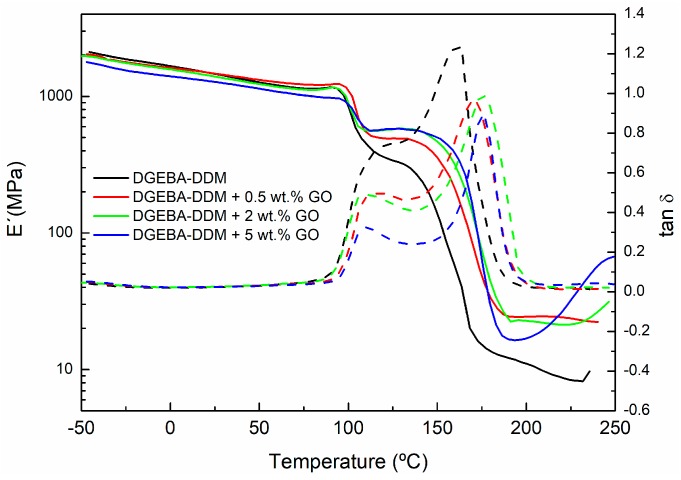
Storage modulus and tan δ of neat epoxy and composites with 0.5, 2, and 5 wt % of GO cured at a temperature of 90 °C.

**Figure 12:**
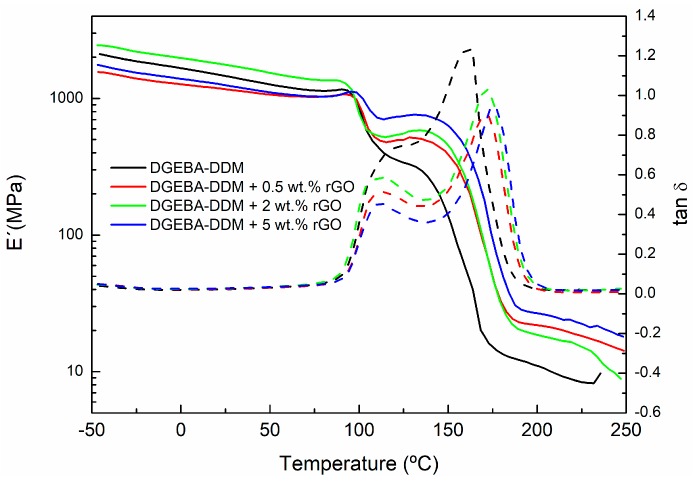
Storage modulus and tan δ of neat epoxy and composites with 0.5, 2 and 5 wt % of rGO cured at a temperature of 90 °C.

Dynamic mechanical measurements are very sensitive to chemical and physical structure of polymeric materials. Therefore, in this work DMTA analysis was used to examine the composite morphology and to measure its glass transition temperatures (*T*_g_). In this way, Figure 11 and Figure 12 show, respectively, the effect of different additive amounts of GO and rGO on storage modulus (E′) and tan δ of graphene based epoxy composites isothermally cured for 4 h at 90 °C. The results shown were obtained with a frequency of 1 Hz.

In the DMTA measurements, the low temperature zone corresponds to the glassy state of the polymer, where the storage modulus is high, decreasing slowly with the increase of the temperature. As temperature increases, free segments begin to move and the excess energy is dissipated as heat. When the temperature reaches around 100 °C, a sharp fall is observed in the storage modulus (E′). This temperature range corresponds to the glass transition region of the epoxy resin cured at 90 °C. In this transition zone, mechanical strength significantly and rapidly decreases. This low glass transition temperature may be explained by the sample preparation process prior to DMTA measurement. During the isothermal curing process at 90 °C the material was only partly cured. Then, as the sample is heated, it enters the glass-transition region (around 100 °C). Thus, the glass transition region (defined by the *T*_g_ of the material) will be determined by the particular cure degree of the sample, i.e., by the curing time and temperature. When the temperature increases above 100 °C the sample continues curing, as the sample was only partly cured at 90 °C. The thermal energy provides enough molecular mobility to reinitiate the curing process, causing a shift in the transition region and an increase in the E′ modulus. As the temperature is raised further still, the reaction finally ceases as the system approaches to full cure. At this point, the modulus decreases again as the material enters the rubbery region. 

Figure 11 and Figure 12 show that the E′ values are higher for all composites after the *T*_g_ of the material compared to neat epoxy. Thus, between 100 and 150 °C the value of E′ modulus increases as the concentration of GO and rGO rises. This increase in the E´ module, observed for both fillers (GO and rGO), indicates a higher crosslinking density for the composites with higher content of GO and rGO. Moreover, in the rubbery state (200–250 °C), the E′ modulus is still higher for both systems modified with GO and rGO compared to neat epoxy. This fact could indicate a better crosslinking network.

In Figure 11 and Figure 12, the evolution of the tan δ with the temperature can also be seen. Tan δ exhibits two peaks: the first one corresponds to the *T*_g_ of the partially-cured material, whereas the second peak corresponds to *T*_g_ of the fully-cured material (*T*_g_∞). These two glass transitions overlap the first one appearing as a shoulder of the second one. Therefore, in order to determine both glass transition temperatures more precisely it is necessary to carry out a deconvolution process, with a symmetrical shape assumed for both transition peaks. Table 4 summarizes the glass transition temperatures determined in this way from the tan δ peaks.

**Figure 13:**
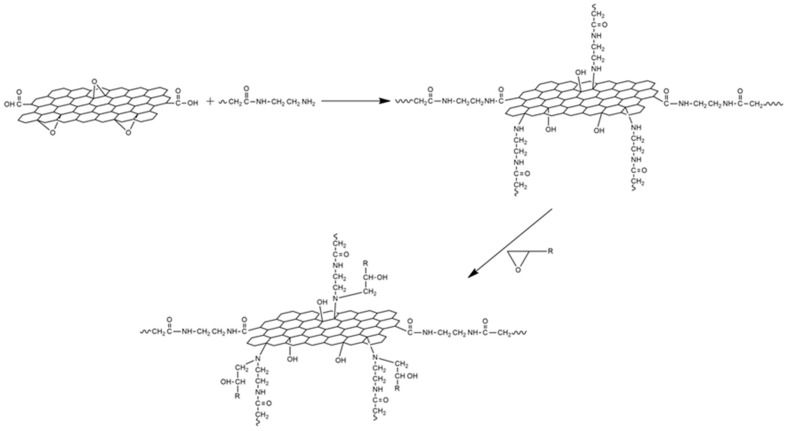
Proposed curing mechanism of GO- and rGO-modified epoxy systems.

The first tan δ peak, corresponding to the *T*_g_ of the initially cured material, does not shift too much with increasing additive content. However, the second tan δ peak, corresponding to *T*_g_ of the fully-cured material, shifts to higher temperatures as the GO and rGO content increase. In the case of GO, the increase in *T*_g_∞ relative to the neat epoxy (sample DGEBA-DDM) can be around 15–20 °C depending the amount of GO used. When rGO is employed as additive, this increase is slightly lower (10–20 °C). The increase in *T*_g_ is often associated with a restriction in molecular motion, a reduction in free volume and a higher degree of crosslinking, indicating significant changes in polymer chain dynamics. Therefore, an increase in *T*_g_∞ would be indicative of good interfacial adhesion between the epoxy matrix and the graphene. The presence of functional groups on the surface of graphene enhances uniform dispersion within resin matrix which in turn leads to creating an interphase zone surrounding GO and rGO fillers due to the chemical interaction between these functional groups on the GO and rGO surfaces with the epoxy. This increased surface area in contact with the epoxy resin, together with the possible formation of covalent bonds between GO and rGO fillers with the epoxy matrix, constrains the chain mobility of the matrix even further and results in a larger *T*_g_∞ shift, so the networks obtained have a higher crosslinking density resulting in an increased rigidity [38]. An example of the proposed mechanism for the interaction of GO and rGO with the epoxy/amine system is shown in Figure 13. 

The shape of the tan δ curve is also related to the structural changes. The lower peak height of tan δ, defined by the amplitude value, for epoxy/GO and epoxy/rGO composites compared to neat epoxy indicates the formation of a higher crosslinked network. Hence, it can be concluded that the composite with 5 wt % of GO have the highest crosslinking density. 

## 4. Conclusions

In this study, the effects of the chemical groups on the graphene surface on the thermo-physical properties of nanocomposites have been analyzed. Initially, GO was prepared from natural graphite with the modified Hummer oxidation method and, thereafter, hydrazine was employed to prepare rGO. The GO and rGO have been thoroughly characterized by FTIR, XPS, and XRD. The results showed the presence of different oxygen functional groups in the GO structure (e.g., carbonyl, epoxy, hydroxyl groups) confirming the successful oxidation of GO. Moreover, a significant removal of oxygen functional groups in rGO was obtained, indicating a partial reduction of the functional groups. 

Thereafter, GO/epoxy and rGO/epoxy nanocomposites were successfully prepared and thoroughly characterized. The DMTA results showed that the addition of GO and rGO causes a significant increase in glass transition temperature and storage modulus in comparison with the neat epoxy. The presence of functional groups on the surface of graphene leads to chemical interaction between these functional groups on the GO and rGO surfaces with the epoxy-amine network. This could contribute to the possible formation of covalent bonds between GO and rGO fillers with the epoxy matrix, constraining the chain mobility of the matrix and resulting in networks with higher crosslinking density and, therefore, with an increased rigidity. Moreover, the XRD and TEM results showed that the oxidation groups on GO are responsible for obtaining composites with improved exfoliation, intercalation, and distribution of the GO sheets in the composites with respect to the rGO-based composites. This better exfoliation can be responsible of the higher transparency observed for GO-modified composites.

## Figures and Tables

**Table 1:** XPS data of GO and rGO.

Cycle	Name	GO				rGO			
		Position	Area	% Conc.	at % rel.	Position	Area	% Conc.	at % rel.
**C**	C–C, C–H	284.6	22,413.7	42.651	69.5	284.5	26,100.5	50.2	81.4
	C1s	285.5	4721.1	8.984		285.4	6893.5	13.3	
	C–O	286.6	4015.9	7.642		286.4	4427.9	8.5	
	O–C=O	288.8	3121	5.939		288.6	3383.1	6.5	
	C1s, sat	291.5	1945.4	3.702		291.2	1292	2.5	
	C1s, sat	295.3	292.8	0.557		294.1	235.2	0.5	
**O**	O1s	532.1	23,890.1	15.515	25.2	531.7	13,405.1	8.8	15.6
	O1s	533.5	14,881.1	9.664		533.2	10,363.7	6.8	
**S**	S2p_3/2_	169.1	3146.3	3.564	5.3	168.7	1015.1	1.162	1.7
	S2p_1/2_	170.3	1573.5	1.782		169.9	507.7	0.6	
**N**	N1s					400.6	1168.8	1.249	1.2

**Table 2:** Measured interplanar spacings converted to lattice constants for graphite.

	(*hkl*)	2θ (°)	*d* (A)	Lattice (A)
c	(002)	26.70	3.336	6.67
	(004)	54.82	1.673	6.69
a	(110)	77.65	1.229	2.46
R	R(101)	43.573	2.075	
	R(012)	46.346	1.958	

**Table 3:** Measured interplanar spacings converted to lattice constants, dispersion, and semi-quantitative analysis for GO and rGO.

Sample	2θ (°)	*d* (A)	Lattice (A)	FWHM	Dispersion	%
GO	24.440	3.64	7.28	13	-	52
	25.425	3.50	7.00	4	3.03–4.14	43
	26.528	3.36	6.72	0.8	3.26–3.46	5
rGO	24.530	3.62	7.24	6	2.92–4.79	41
	26.233	3.39	6.78	2	3.15–3.67	57
	26.528	3.36	6.72	0.8	3.26–3.46	<2

**Table 4:** Glass transition temperatures of neat epoxy system and composites with 0.5, 2, and 5 wt % of GO and rGO cured at a temperature of 90 °C.

Sample	Peak 1 (°C)	Peak 2 (°C) *T*_g_∞
DGEBA-DDM	110.8	154.9
DGEBA-DDM + 0.5 wt % GO	112.7	170.2
DGEBA-DDM + 2 wt % GO	105.4	176.3
DGEBA-DDM + 5 wt % GO	112.0	176.3
DGEBA-DDM + 0.5 wt % rGO	105.2	162.4
DGEBA-DDM + 2 wt % rGO	107.8	162.4
DGEBA-DDM + 5 wt % rGO	113.7	173.9
